# Exploring the role of clinical and demographic characteristics on the effects of virtual reality cognitive behavioral therapy for psychosis: A moderator analysis

**DOI:** 10.1111/acps.13713

**Published:** 2024-06-09

**Authors:** M. Berkhof, E. C. D. van der Stouwe, R. M. C. A. Pot‐Kolder, M. van der Gaag, W. Veling, C. N. W. Geraets

**Affiliations:** ^1^ University Medical Center Groningen University of Groningen Groningen the Netherlands; ^2^ Department of Clinical Psychology VU University Amsterdam the Netherlands; ^3^ Research Department Parnassia Psychiatry Institute The Hague the Netherlands

**Keywords:** cognitive behavioral therapy, paranoia, Psychosis, virtual reality

## Abstract

**Background:**

Virtual Reality cognitive behavioral therapy (VR‐CBT) has proven to be an effective treatment method for paranoia and anxiety in psychosis. However, it is unknown, which individuals benefit most from VR‐CBT. Previous studies examined factors affecting the treatment effect of regular CBTp, including illness duration, paranoia, depression, and pre‐therapy avoidance behaviors, but results are inconsistent. The study aims to investigate the factors that influence the effectiveness of VR‐CBT.

**Methods:**

A total of 95 participants with a psychotic disorder and at least moderate paranoia (GTPS >40) were included in this explorative study. Data were collected as part of a multicenter randomized controlled trial in which participants were assigned to VR‐CBT or treatment as usual (TAU). The VR‐CBT group received 16 sessions of individual treatment. A moderator analysis was conducted to examine the influence of baseline demographic (age, gender, and education level) and clinical characteristics (duration of illness, paranoia, anxiety, depression, safety behavior, self‐esteem, and social functioning) on treatment effects of paranoia and anxiety as measured with questionnaires and the experience sampling method (ESM) directly after treatment (12 weeks after baseline).

**Results:**

More use of safety behavior at baseline resulted in greater benefits of VR‐CBT on paranoid ideation and ESM paranoia. A higher age was associated with greater benefits of VR‐CBT on social anxiety but not paranoia outcomes. There was no consistent evidence of moderation by any of the other sociodemographic or clinical variables for paranoid ideation and social anxiety.

**Conclusions:**

Our findings suggest that a diverse spectrum of patients, with different backgrounds and symptom severity may be able to benefit from VR‐CBT. VR‐CBT can be recommended to a broad spectrum of patients with psychotic disorders, and particularly those with high levels of safety behaviors, including severe avoidance, seem to benefit more.

**Significant outcomes**

- More safety behavior use at baseline was associated with greater benefits of VR‐CBT on paranoid ideation and anxiety.
- Higher age was related to greater benefits of VR‐CBT on social anxiety, but not paranoia.
- Results suggest a broad spectrum of patients with psychotic disorders can profit from VR‐CBT.

**Limitations**

- The study lacked an active control group.
- The sample may be somewhat selective, as the most avoidant patients probably did not participate.
- Due to the explorative nature of the moderation analyses, we did not correct for multiple testing.

## 1 INTRODUCTION

Paranoid delusions, which involve the unfounded belief that others are attempting to cause harm, are prevalent among 70% of patients with psychotic disorders.^1^, ^2^ These delusions are associated with distress, anxiety, depression, and suicidal ideation, as well as hospitalization and impaired social functioning.^1^, ^3^, ^4^, ^5^ A recent study showed that Virtual Reality cognitive behavioral therapy for psychosis (VR‐CBTp) was effective in reducing paranoia.^6^ However, it is unknown, which individuals benefit most from VR‐CBT. Therefore, we investigated the moderating effects of clinical and demographic factors on the treatment effect of VR‐CBT.

Previous studies have investigated how clinical characteristics affect the efficacy of regular CBTp. For example, a shorter illness duration has been related to a better treatment response.^7^, ^8^, ^9^ One study, however, indicated that duration of illness did not predict the efficacy of CBTp.^7^ Furthermore, several studies have investigated the relationship between symptom levels at the start of the therapy and subsequent response to CBTp.^7^, ^8^, ^10^, ^11^, ^12^, ^13^, ^14^, ^15^ Some studies found that patients with lower levels of self‐esteem, depression, negative symptoms, and impairment of social functioning showed more improvement in their positive symptoms after receiving CBTp.^9^, ^10^, ^16^ In contrast, other research has shown that more severe positive symptoms (e.g., paranoid delusions) at baseline were related to a better response to CBTp.^14^, ^16^ Yet another study indicated that the severity of anxiety, depression, and positive symptoms (e.g., paranoid delusions) at baseline did not serve as a predictor for the efficacy of CBTp.^7^ Thus, it remains unclear how certain clinical characteristics of patients affect the effectiveness of CBTp.

Furthermore, several studies have explored the association between sociodemographic factors and treatment response, suggesting a better treatment response related to being female,^11^, ^16^, ^17^ being older,^18^, ^19^ or having a higher level of education.^16^, ^19^ However, one study indicated that age did not predict the efficacy of CBTp.^7^ Additionally, a recent meta‐analysis of individual‐participant data found that the efficacy of CBTp was not influenced by age, gender or symptom severity.^20^ In sum, findings regarding the role of sociodemographic factors in the efficacy of regular CBTp have been inconsistent.

Virtual Reality cognitive behavioral therapy for psychosis is a relatively new form of CBTp where VR‐technology is used as a tool to create engaging, controlled, tailored and immersive simulations of anxiety‐provoking real‐life situations to help patients learn and practice new behavior and coping skills to manage their symptoms and problems. Recent VR‐CBTp studies have shown promising results, with large effect sizes (Cohen's *d =* 0.86 to 1.49).^6^, ^21^, ^22^ Specifically, these studies found that using VR‐CBT, with a focus on eliminating safety behaviors, led to significant reductions in delusions, distress, and paranoia in daily life.^6^, ^21^, ^22^ In other words, VR‐CBTp can be effective in helping individuals overcome delusions and associated symptoms.

While there is emerging evidence supporting the effectiveness of VR treatments, there is still a lack of knowledge regarding the specific patient characteristics that may influence treatment outcomes. A recent meta‐analysis on virtual reality applications in the treatment of anxiety disorders demonstrated that mean age of participants did not moderate treatment outcome.^23^ With regard to VR‐CBTp specifically, one recent study found automated VR‐CBTp to be effective in patients with agoraphobia and avoidance safety behaviors.^22^ Specifically, those with severe avoidance at the start of the treatment experienced significant and meaningful benefits up until the 6‐month follow‐up. Furthermore, the severe avoidance group demonstrated broader positive outcomes from VR therapy including quality of life.^22^ However, this was a fundamentally different type of intervention compared to our VR‐CBTp protocol, which was not automated, but entailed close therapist involvement and engagement.^6^, ^24^ Although the outcomes of this research^22^ shed a first light on patient characteristics moderating the effectiveness of VR‐CBTp for paranoia, more research is needed to identify which patients can benefit most from VR‐CBTp.

To summarize, there is evidence supporting the efficacy of (VR)‐CBTp in treating paranoia, but it remains unclear how clinical characteristics and sociodemographic factors influence treatment outcomes. To extend the current knowledge, we conducted a moderator analysis to explore the impact of demographical characteristics (i.e., age, gender, and education level) and clinical characteristics at the start of treatment (i.e., duration of illness, paranoia, anxiety, depression, safety behavior, self‐esteem, and social functioning) on treatment effects, using data from the randomized controlled trial (RCT) called ‘Virtual Reality Exposure Therapy for Psychosis’ (VRET.P).^6^

## 2 METHODS

### 2.1 Study design & participants

This study was a multicenter RCT comparing VR‐CBT plus treatment as usual (TAU) with TAU.^6^ Assessments were conducted by research assistants blinded to treatment allocation at baseline, post‐treatment (three months after baseline), and six month follow‐up. A total of 116 participants were randomized after the baseline assessment. In the current study, participants were only included if they completed the post‐treatment measure, which 10 did not. Furthermore, participants from the VR‐CBT group were included only if they completed all 16 sessions. Eleven patients did not complete VR‐CBT. Four never started (0 sessions), two stopped after session 1 (due to feeling too afraid and having no time), two stopped after session 2 (unwilling to travel to therapy, nausea) one dopped‐out after 3 session (unable to attend therapy sober), and two found the head‐mounted display too uncomfortable and stopped after session 5 and 6. This resulted in a total sample of 95 participants.

Participants were recruited from seven mental health centers. To be included in the study, participants had to be between 18 and 65 years old, have a DSM‐IV diagnosis of a psychotic disorder, avoidance of specific situations (shops, streets, public transport, bars or restaurants), and paranoid ideation in the past month (score of at least 40 on the Green Paranoid Thoughts Scale^25^). Exclusion criteria included a history of epilepsy, IQ below 70, and insufficient command of the Dutch language. The study protocol was approved by the medical ethical committee of VU University Medical Centre Amsterdam (METC number NL37356.058.12), and written informed consent was obtained from all participants. Participants received 30 euro compensation for each completed assessment.

### 2.2 Intervention

**VR‐CBT** The VR‐CBT intervention consisted of 16 one‐hour individual therapy sessions conducted by trained psychologists. The psychologists had at least completed a basic CBT training and received two days of specific VR‐CBT training. Therapists attended monthly group supervision.

The treatment protocol aimed to reduce paranoia and improve social participation. The therapy sessions followed a structured treatment plan outlined in a manual. The initial sessions involved setting personal treatment goals and introducing participants to the VR system. In the subsequent sessions, participants spent 40 min practicing within VR environments. It incorporated evidence‐based CBT elements, such as setting treatment goals, exposures, behavioral experiments, reducing safety behaviors, and attention strategies. However, instead of conducting these exercises and experiments in real‐world settings, they were done in VR environments (a bus, supermarket, bar and street environment were available in VR).

In the VR environment, participants were able to navigate using a Logitech F310 Gamepad. They utilized a Sony HMZ‐T1/T2/T3 Head Mounted Display. The head rotation was tracked with a 3DOF (degree of freedom) tracker. Therapists could adjust the number of human avatars present, ranging from 0 to 40. They could also customize the avatars' characteristics, such as sex and ethnicity, and determine their responses toward the patient, including neutral or hostile behavior and eye contact. Additionally, therapists had the ability to make the avatars deliver pre‐recorded sentences. This level of control allowed the therapists to create personalized treatment exercises tailored to the individual patient, aligning with their specific paranoid fears. No homework was assigned as part of the therapy. More comprehensive details of the intervention have been previously published.^6^, ^24^

**TAU** The participants in the TAU group received the standard treatment, which could include antipsychotic medication and consultations and/or supportive counseling provided by psychiatrists, psychiatric nurses, or social workers. However, the TAU group was not permitted to receive any form of psychological therapy specifically focused on improving social participation during the trial.

### 2.3 Measures

**Paranoid ideation** within the past month were assessed with the *Green Paranoid Thoughts Scale* (GPTS).^25^ The GPTS is a self‐report questionnaire that consists of two subscales each including 16 items. Part A of the scale assesses paranoid delusions related to social reference, while Part B measures experiences of social persecution. Participants rate their responses on a five‐point Likert scale. Both scales have good internal consistency and validity.^25^

**Momentary paranoia** in daily life was assessed with *the Experience Sampling Method* (). The ESM method used in this study was the PsyMate electronic device,^26^, ^27^ which has high ecological validity.^28^ Participants were prompted 10 times a day over 6 days to provide self‐assessments on a seven‐point Likert scale within 15 min of each prompt. The ESM was conducted at baseline and post‐treatment. To be included in the analysis, participants had to complete at least one‐third of these assessments, totaling a minimum of 20 measurements. The ESM items used in the study were derived from previous research.^29^ Factor analysis confirmed the momentary paranoia factor for three items, which constituted the three‐item momentary paranoia subscale calculated as the mean score of 1. “I feel that others might hurt me”; 2. “I feel that others dislike me”; and 3. “I feel suspicious”. An average score was calculated per participant per assessment period for analyses.

**Momentary anxiety with others** in daily life was also measured by ESM with the PsyMate device.^26^, ^27^ Scores on the item “I feel anxious” while also indicating being in the presence of other people was used to establish the average momentary anxiety in company of others.

**Social Interaction anxiety** symptoms were assessed with the *Social Interaction Anxiety Scale* (SIAS).^30^ The SIAS consists of 19 items that assess the tendency to fear and avoid social situations. The scale demonstrated high levels of internal consistency and test–retest reliability.^30^

**Safety behaviors** were assessed by the *Safety Behaviors Questionnaire – persecutory delusions* (SBQ).^15^ The purpose of the SBQ is to assess the use of safety behaviors through a semi‐structured interview where patients are asked to rate the frequency of their safety behaviors over the past month using a four‐point scale. The SBQ has demonstrated high inter‐rater reliability, acceptable test–retest reliability, and satisfactory validity.^15^

**Depression** was assessed by the *Beck Depression Inventory – II* (BDI‐II)^30^ which is a self‐report instrument comprising 21 items that evaluate the severity of depressive symptoms experienced by an individual in the past two weeks. The BDI‐II has exhibited adequate psychometric properties.^13^

**Self‐esteem** was evaluated using the *Brief Core Schema Scales* (BCSS),^31^ a self‐report measure comprising 24 items that assess an individual's beliefs about themselves and others on a 5‐point rating scale. The BCSS has demonstrated good psychometric properties, including construct validity.^31^

**Social Functioning** was measured by the *Social and Occupational Functioning Assessment Scale* (SOFAS)^32^ which is a tool used in clinical settings to assess an individual's level of social and occupational functioning. It is a rating scale that ranges from 0 to 100, with higher scores indicating better functioning. The psychometric properties of the SOFAS are generally considered to be good, including inter‐rater reliability and convergent validity.^32^

### 2.4 Statistical analyses

A sensitivity analysis was performed for the moderation analyses. The sensitivity analysis showed that a regression with four predictors, 95 participants, an alpha of 0.05 and a power of 0.80 can detect an effect size f2 of 0.08, which is a small to medium effect.

First, linear regression analyses were used to evaluate the general treatment effects for the outcome measures paranoid ideation (GPTS), social anxiety (SIAS), momentary paranoia (ESM) and momentary anxiety with others (ESM). Treatment group and the baseline outcome measure were predictors in the analyses.

Next, separate linear regression analyses were used to evaluate whether sociodemographic (age, gender, and education level) and clinical variables (duration of illness, paranoia, anxiety, depression, safety behavior, self‐esteem, and social functioning) moderated the effects of VR‐CBT versus TAU in reducing paranoid ideation and social anxiety at post‐treatment. These moderators were chosen a priori before commencing the analyses. Treatment group, the baseline outcome measure (respectively paranoia or social anxiety), the moderator, and the interaction between group and the moderator were the independent variables in the analyses.

Significant moderator effects were visualized with post‐hoc probing using the pick‐a‐point approach.^33^ This approach gives estimates of the outcome measure for values of the moderator equal to one standard deviation below the sample mean and one standard deviation above the mean, representing low, medium and high levels of the moderator. All analyses were carried out per moderator. A *p*‐value of less than 0.05 was used as the significance level. SPSS version 28 (IBM Corp., Armonk, NY) was used for these explorative moderator analyses.

## 3 RESULTS

In total 95 participants were included in the analyses. Table 1 presents the baseline information of the participants. The VR‐CBT and TAU group did not differ significantly on any of the sociodemographic and clinical characteristics at baseline. Regression analyses showed significant treatment effects (effect of group) for paranoid ideation and momentary paranoia in favor of the VR‐CBT intervention, see Table 2.

**TABLE 1:** Sociodemographic and clinical characteristics.

	VR‐CBT	TAU
	M (SD)	M (SD)
	*n* = 42	*n* = 53
Age, y	38.2 (10.1)	40.2 (9.9)
Male (%)	70%	70%
Level of education (%)		
No/primary	26%	26%
Vocational	28%	42%
Selective secondary	19%	15%
Higher	26%	17%
Clinical diagnosis (%)		
Schizophrenia	86%	91%
Schizoaffective disorder	2%	2%
Delusional disorder	2%	0%
Psychotic disorder no's.	10%	6%
Duration of illness	14.9 (11.3)	15.3 (9.2)
Baseline paranoid ideation	83.6 (32.5)	78.5 (31.5)
Baseline social anxiety	44.3 (13.0)	42.8 (15.0)
Baseline depressive symptoms	17.1 (9.2)	17.3 (9.8)
Baseline safety behavior	29.3 (14.6)	25.1 (15.3)
Baseline self‐esteem positive	8.2 (5.4)	7.1 (4.4)
Baseline self‐esteem negative	5.8 (4.9)	5.4 (4.5)
Baseline social functioning	49.1 (7.6)	49.8 (8.4)

**TABLE 2:** Means, standard deviations and test results over time.

	VR‐CBT		TAU		Treatment effect of group		
	Baseline	Post	Baseline	Post	b(SE)	p	95% CI
	N = 42		N = 53				
Paranoia ideation	83.6 (32.5)	66.10 (32.3)	78.5 (31.5)	76.9 (31.9)	−14.7 (4.4)	0.001	−23.3: −6.0
Social anxiety	44.3 (13.0)	37.2 (13.8)	42.8 (15.0)	39.2 (15.7)	−3.1 (2.2)	0.16	−7.37: 1.19
	N = 41		N = 48				
Momentary paranoia	3.08 (1.34)	2.73 (1.35)	3.21 (1.50)	3.31 (1.61)	−0.46 (0.15)	0.003	−0.77: −0.16
	N = 38		N = 44				
Momentary anxiety	3.06 (1.18)	2.64 (1.15)	3.20 (1.49)	3.18 (1.60)	−0.40 (0.20)	0.14	−1.05: 0.24

### 3.1 Moderation of paranoid ideation (GPTS)

**FIGURE 1:**
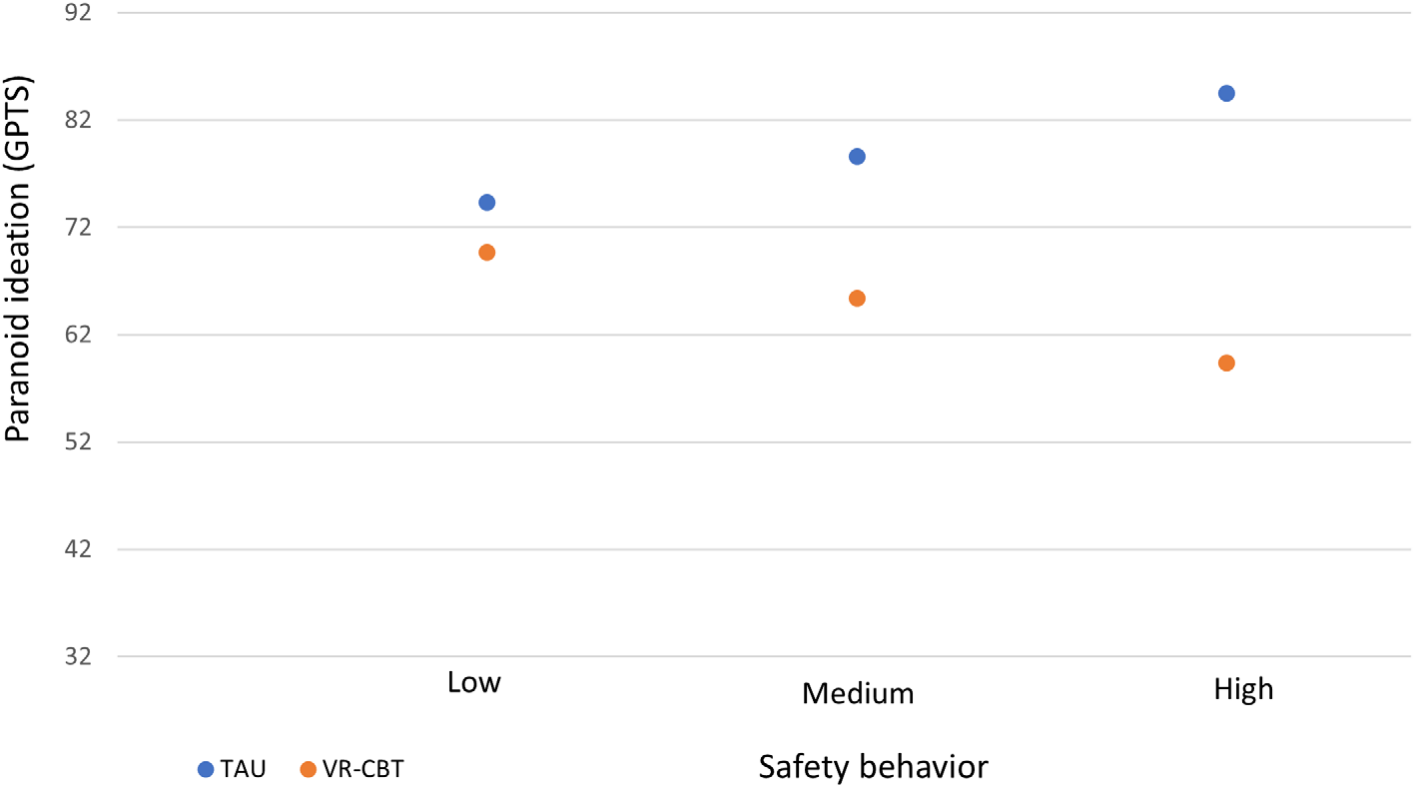
Post‐hoc probing effect of baseline safety behavior on the effect of treatment on paranoid ideation (GPTS, post‐treatment scores).

All outcomes for sociodemographic and clinical variables as potential moderators of treatment effects are shown in Table 3. Baseline safety behavior (as assessed by the SBQ) significantly moderated the relation between group and paranoid ideation at post‐treatment while controlling for baseline paranoid ideation. More specifically, participants who showed more safety behavior at baseline resulted in greater treatment benefits of VR‐CBT at post‐treatment (3 months after baseline), than participants with high safety behavior who received TAU. Post‐hoc probing of the effect (see Figure 1) revealed that for participants with low safety behavior (SBQ = 12) there was no significant effect of group on paranoid ideation (mean score at post‐treatment *M*_post vr‐cbt_ = 69.7 and *M*_post tau_ = 74.5, *b* = −4.6, CI [−17.08: 7.82], *p* = 0.46). For medium safety behavior (SBQ = 25) the difference was significant (*M*_post vr‐cbt_ = 65.4 and *M*_post tau_ = 78.6, *b* = −13.2, CI [−21.92: −4.50], *p* = 0.003) and for high safety behavior (SBQ = 43) differences were largest (*M*_post vr‐cbt_ = 59.4 and *M*_post tau_ = 84.5, *b* = −25.1, CI [−37.55: −12.65], *p* = 0.0001). There was no evidence of moderation of treatment effects by any of the other sociodemographic or clinical variables for paranoid ideation.

**TABLE 3:** Results of the moderator analyses.

	Interaction group X moderator					
Moderator	b(SE)	P	95% CI	b(SE)	P	95% CI
	Paranoid ideation GPTS (*n* = 95)			Momentary paranoia ESM (*n* = 89)		
Age	0.69 (0.44)	0.12	−0.19: 1.56	0.00 (0.02)	0.96	−0.03: 0.03
Gender	−0.85 (9.62)	0.93	−19.96: 18.26	0.34 (0.33)	0.31	−0.32: 1.01
Level of education	3.33 (4.06)	0.41	−4.73: 11.39	0.08 (0.14)	0.57	−0.20: 0.37
Illness duration	0.22 (0.43)	0.61	−0.63: 1.06	0.02 (0.02)	0.25	−0.01: 0.05
Baseline paranoid ideation	−0.02 (0.14)	0.86	−0.30: 0.25	−0.01 (0.00)	0.18	−0.02: 0.00
Baseline social anxiety	−0.05 (0.32)	0.88	−0.68: 0.59	−0.01 (0.01)	0.31	−0.03: 0.01
Baseline depressive symptoms	−0.25 (0.47)	0.59	−1.19: 0.68	−0.03 (0.02)	0.12	−0.06: 0.01
Baseline safety behavior	−0.66 (0.29)	**0.03** *	−1.24: −0.08	−0.03 (0.01)	**0.005** *	−0.05: −0.01
Baseline self‐esteem positive	0.10 (0.92)	0.91	−1.72: 1.93	0.06 (0.03)	0.06	−0.00: 0.12
Baseline self‐esteem negative	0.22 (0.93)	0.81	−1.63: 2.07	−0.06 (0.03)	0.07	−0.12: 0.00
Baseline social functioning	−0.06 (0.56)	0.92	−1.18: 1.06)	−0.01 (0.02)	0.69	−0.05: 0.03

	Social anxiety SIAS (*n* = 95)			Momentary anxiety with others ESM (*n* = 82)		
Age	−0.41 (0.21)	**0.05** *	−0.83: 0.00	−0.01 (0.02)	0.51	−0.05: 0.03
Gender	−4.73 (4.64)	0.31	−13.95: 4.49	0.28 (0.40)	0.49	−0.52: 1.08
Education	2.76 (2.01)	0.17	−1.23: 6.74	0.06 (0.17)	0.73	−0.28: 0.40
Illness duration	−0.02 (0.21)	0.94	−0.44: 0.41	0.02 (0.02)	0.40	−0.02: 0.06
Baseline paranoid ideation	0.04 (0.07)	0.53	−0.09: 0.18	−0.01 (0.01)	0.24	−0.02: 0.00
Baseline social anxiety	−0.18 (0.16)	0.25	−0.50: 0.13	−0.02 (0.01)	0.15	−0.04: 0.01
Baseline depressive symptoms	0.01 (0.23)	0.96	−0.45: 0.48	−0.05 (0.02)	**0.01** *	−0.08: −0.01
Baseline safety behavior	−0.00 (0.15)	0.99	−0.30: 0.29	−0.03 (0.01)	**0.01** *	−0.06: −0.01
Baseline self‐esteem positive	−0.52 (0.44)	0.24	−1.41: 0.36	0.05 (0.04)	0.15	−0.02: 0.13
Baseline self‐esteem negative	0.33 (0.45)	0.46	−0.56: 1.23	−0.07 (0.04)	0.08	−0.14: 0.01
Baseline social functioning	0.13 (0.28)	0.64	−0.42: 0.68	0.02 (0.02)	0.48	−0.03: 0.06

* Marks significant effects at *p* < 0.05.

### 3.2 Moderation of momentary paranoia (ESM)

**FIGURE 2:**
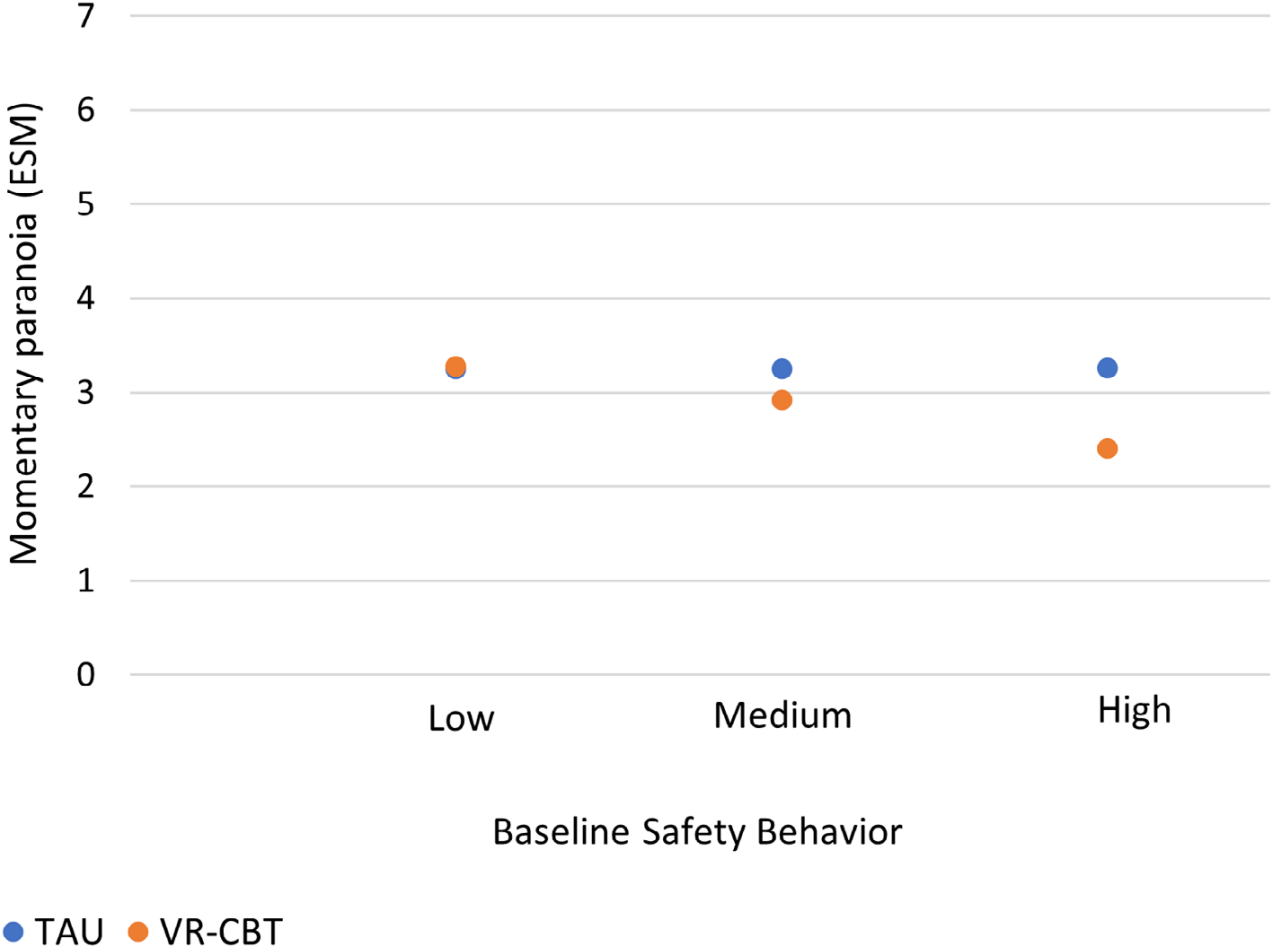
Post‐hoc probing effect of safety behavior on the effect of treatment on momentary paranoia (ESM, post‐treatment scores).

Momentary paranoia was also moderated by baseline safety behavior when controlling for baseline momentary paranoia. Similar to paranoid ideation, participants with more baseline safety behavior had greater benefit from VR‐CBT by post‐treatment, than participants with high safety behavior who received TAU. Post‐hoc probing (see Figure 2) showed that for participants with low safety behavior (SBQ = 12) there was no significant effect of group on momentary paranoia (mean score at post‐treatment *M*_post vr‐cbt_ = 3.27 and *M*_post tau_ = 3.25, *b* = 0.02, CI [−0.40: 0.44], *p* = 0.91). For medium safety behavior (SBQ = 25) the difference was significant (*M*_post vr‐cbt_ = 2.92 and *M*_post tau_ = 3.25, *b* = −0.33, CI [−0.63: −0.03], *p* = 0.03) and for high safety behavior (SBQ = 43) differences were largest (*M*_post vr‐cbt_ = 2.40 and *M*_post tau_ = 3.26, *b* = −0.86, CI [−1.28: −0.43], *p* = 0.0001).

### 3.3 Moderation of social interaction anxiety (SIAS)

Social interaction anxiety was moderated by age only, when controlling for baseline social interaction anxiety. Participants who were older gained more from VR‐CBT than from TAU compared to younger participants. Probing showed that relatively young patients with an average age of 28 (*M*_post vr‐cbt_ = 41.5 and *M*_post tau_ = 40.7, *b* = 0.7, CI [−5.28: 6.82], *p* = 0.80) or medium aged participants (i.e., 39 years old) (*M*_post vr‐cbt_ = 36.2 and *M*_post tau_ = 39.8, *b* = −3.6, CI [−7.75: 0.48], *p* = 0.08) did not benefit significantly more from the VR‐CBT treatment than from TAU. Whereas relatively older participants (i.e., one standard deviation above the mean age; 48 years) did (*M*_post vr‐cbt_ = 31.7 and *M*_post tau_ = 39.0, *b* = −7.4, CI [−12.90: −1.82], *p* = 0.001). Post hoc checks for paranoid ideation showed that an opposite (non‐significant) effect of age was seen for paranoid ideation.

### 3.4 Moderation of momentary anxiety with others (ESM)

Safety behavior and depression symptoms moderated momentary anxiety in the company of others. Similarly to the paranoia outcomes, patients with more safety behavior benefited more from VR‐CBT than from TAU. For low (*M*_post vr‐cbt_ = 3.02 and *M*_post tau_ = 2.96, *b* = 0.06, CI [−0.45: 0.57], *p* = 0.81) and medium safety behavior (*M*_post vr‐cbt_ = 2.78 and *M*_post tau_ = 3.11, *b* = −0.32, CI [−0.67: 0.04], *p* = 0.99) group was not predictive of the momentary anxiety at post treatment, but for high levels it was (*M*_post vr‐cbt_ = 2.40 and *M*_post tau_ = 3.33, *b* = −0.93, CI [−1.46: −0.40], *p* < 0.001).

Concerning depressive symptoms, the treatment effect was not significantly predictive for people with low (for BDI = 8, *M*_post vr‐cbt_ = 2.84 and *M*_post tau_ = 2.84, *b* = −0.00, CI [−0.49: 0.49], *p* = 0.99) and medium levels (BDI = 15.5, *M*_post vr‐cbt_ = 2.72 and *M*_post tau_ = 3.07, *b* = −0.35, CI [−0.71: 0.01], *p* = 0.06) of depressive symptoms, but treatment did influence the effect of people with high levels (*M*_post vr‐cbt_ = 2.55 and *M*_post tau_ = 3.39, b = −0.83, CI [−1.32: −0.34], *p* = 0.001) as shown by post‐hoc probing, with people benefitting more in the VR‐CBT group.

## 4 DISCUSSION

We aimed to explore the impact of demographical and clinical characteristics on the effect of VR‐CBT on paranoid ideation and social anxiety. This study showed that more use of safety behaviors at start of treatment resulted in greater improvements in paranoia and social anxiety after VR‐CBT compared to TAU. Further, older age was a predictor of better VR‐CBT outcome on social interaction anxiety but not in any of the other measures. No moderation effect of gender and education level or other clinical characteristics (i.e., duration of illness, paranoid ideation anxiety, social anxiety, depression, self‐esteem, and social functioning) was found.

This study selected patients with excessive avoidance behavior and paranoia. The VR‐based CBT targeted the dropping safety behaviors and exposure to the anticipated threat. It was successful in this and the patients with the highest levels of safety behaviors benefitted most. These findings align with the outcomes of the automated VR therapy of Freeman and colleagues, wherein patients with the most severe avoidance behaviors, such as the fear of walking alone on the street, showed the most substantial improvements from the treatment.^22^ A crucial element in both VR‐CBT and automated VR therapy are behavioral interventions such as exposure and behavioral experiments during which patients are explicitly encouraged to drop safety behavior. These behavioral interventions targeted at dropping safety behavior enabled new learning experiences, that is, learning that one is safer than one had thought. However, the current VR‐CBT differed in form from VR therapy by Freeman and colleagues, because VR therapy utilized a virtual coach, whereas the current VR‐CBT was delivered by a therapist, enabling a therapeutic relationship and highly personalized feedback. Similarly to the current study, the automated VR therapy study also found no evidence of treatment moderation effects on paranoia by any other clinical and demographic variables such as age and gender.^22^

We did find that older age predicted a better outcome in VR‐CBT on social interaction anxiety, as well as higher depressive symptoms for momentary social anxiety. However, it should be noted that for none of the other outcomes depression or age were predictive, and that the GPTS paranoid ideation outcome showed findings in the opposite direction for age. These inconsistent findings of depression and age as moderators of VR‐CBT treatment effects are in line with prior inconsistent findings for regular CBT,^22^ which may somewhat question the predictive value of these moderators. Indeed, the lack of clear moderation effects other than safety behavior are broadly consistent with a meta‐analysis of individual‐participant data (IPD) demonstrating that demographic and clinical variables do not significantly impact treatment outcome in CBT for psychosis.^20^ This indicates that a broad spectrum of patients (e.g., male, female, shorter illness duration, chronic) may benefit from VR‐CBT.

The question why for some people VR‐CBTp does not work remains largely unanswered. Possibly more specific individual factors are important for the treatment response, such as personal preferences,^34^ the working alliance,^35^ or feelings of presence in VR.^36^ Thus further investigation is warranted to answer questions, such as for whom, when and how VR therapy works best. To enable this, research should be specifically designed a priori to study potential influencing factors. Also, qualitative research is needed, to better understand the nuances and parameters of effectiveness of VR‐CBT.

Based on our current findings the question arises why people showing a lot of safety behavior benefit most. An explanation could be related to limitations of the software used in the study. There were just four environments available, and participants with less generalized avoidance may have benefited less. An alternative explanation is that with VR‐based CBT, treatment can be directly focused at behavioral components such as reducing avoidance and dropping safety behaviors within the therapy first sessions. With the possibilities to tailor VR sessions to individual needs and the complete control over what happens in VR, the threshold for engaging in exposure and behavioral experiments is lower. Patients can directly experience that they are safer than they thought and it helps them learning that they can tolerate anxiety. These findings align with the cognitive model for paranoia,^1^ wherein the treatment goal is to drop safety behaviors and to experience that threatful events are not occurring as anticipated. Thus, VR‐based CBT targeting the dropping of safety behaviors is able to diminish paranoid ideation and social anxiety.

### 4.1 Strengths & limitations

Strengths of this study include that the dataset was derived from a multicenter high‐quality RCT,^6^ conducted across seven mental health centers. The meticulous execution of the protocol and sustained high‐fidelity expert supervision guaranteed high quality VR‐CBT. This has provided us with valuable insights, allowing a comprehensive understanding of the patient profiles that derive the most significant benefits from VR‐CBT.

This study also had some limitations, which may have affected the results. First only 95 participants of the 116 participants were included in the analyses. However, we do not expect that the withdrawal of participants had a substantial impact on our findings. Examination of the primary randomized controlled trial^6^ indicated no significant differences in baseline paranoid ideation or safety behaviors between participants who completed the VR‐CBT and those who dropped out. Additionally, no disparities were observed in baseline paranoid ideation or safety behavior between participants who abstained from follow‐up measurements and those who completed the entire measurement protocol. Second, the study design lacked an active control group, such as CBT with in vivo exposure. Therefore, the possibility of a dose‐effect related to therapeutic interactions cannot be eliminated. Third, the long‐term effects of safety behavior as moderator in VR‐CBT remain unknown, because we only looked at the effect directly after treatment. Fourth, a potential ceiling effect indicates that individuals with moderate or low level of symptoms had limited room for improvement while individuals with higher initial psychotic symptoms had greater potential for improvement, acknowledging that severe symptoms inherently provide more capacity for improvement. Finally, this study was an explorative study with secondary analyses of an RCT. Due to the explorative nature of the moderation analyses we did not correct for multiple testing.^37^ As a consequence, the type 1 error (probability of falsely concluding that there is a significant effect when there is no effect) is relatively high in this study. Therefore results need to be interpreted with caution and replication studies with confirmatory analyses are needed.

### 4.2 Conclusion & future research

Sociodemographic and clinical variables moderated treatment effects of VR‐CBT only to a limited degree, thus a broad spectrum of patients may be able to benefit from VR‐CBT. As VR‐CBT did benefit patients characterized by the use of severe safety behaviors most, this intervention should be offered in particular to those patients with high levels of safety behaviors. Furthermore, the results of the current study further emphasize the crucial role of behavioral interventions in (VR)CBT for paranoid ideations.^38^

Future research will need to replicate our findings. Also, other clinical factors that have been demonstrated to influence the effectiveness of CBTp should be investigated as potential moderators of VR‐CBT effects, such as negative symptoms^8^, ^16^ and clinical insight.^7^, ^16^ Furthermore, clarity on the differential efficacy of VR‐CBT compared to traditional CBTp is crucial in determining the preferred treatment for specific individuals. Several RCTs comparing these two treatments for paranoid ideations are currently ongoing.^39^, ^40^

## AUTHOR CONTRIBUTORS

W. Veling, M. van der Gaag, and R.M.C.A. Pot‐Kolder designed the study. The study was supervised by W. Veling and M. van der Gaag. C.N.W. Geraets and R.M.C.A. Pot‐Kolder contributed with acquisition, administrative and technical support. M. Berkhof managed the literature searches. C.N.W. Geraets conducted the statistical analysis. M. BerkhofB wrote the first draft of the manuscript, which was critically revised by C.N.W. Geraets. E.C.D. van der Stouwe and W. Veling. All authors contributed to and have approved the final manuscript.

## FUNDING INFORMATION

Funders had no involvement in the study design; in the collection, analysis and interpretation of data; in the writing of the report; nor in the decision to submit the article for publication.

## CONFLICT OF INTEREST STATEMENT

All authors declare that they have no conflicts of interest.

### PEER REVIEW

The peer review history for this article is available at https://www.webofscience.com/api/gateway/wos/peer-review/10.1111/acps.13713.

## Data Availability

The data that support the findings of this study are available on request from the corresponding author. The data are not publicly available due to privacy or ethical restrictions.
